# Influence of cast change interval in the Ponseti method: A systematic review

**DOI:** 10.1371/journal.pone.0199540

**Published:** 2018-06-22

**Authors:** R. B. Giesberts, M. C. van der Steen, P. G. M. Maathuis, A. T. Besselaar, E. E. G. Hekman, G. J. Verkerke

**Affiliations:** 1 Department of Biomechanical Engineering, University of Twente, Enschede, the Netherlands; 2 Department of Orthopaedic Surgery, Catharina Hospital Eindhoven, Eindhoven, the Netherlands; 3 University of Groningen, University Medical Center Groningen, Department of Orthopaedic Surgery, Groningen, the Netherlands; 4 Orthopaedic Center Máxima, Máxima Medical Center, Eindhoven, the Netherlands; 5 University of Groningen, University Medical Center Groningen, Department of Rehabilitation Medicine, Groningen, the Netherlands; University Hospital Jena, GERMANY

## Abstract

**Background:**

Clubfeet are commonly treated using the Ponseti method. This method involves weekly manipulation and casting which gradually corrects the position of the foot. However, the reasons for following a weekly interval are not clear.

**Question / Purpose:**

The aim is to investigate the influence of the cast change interval on treatment outcomes in the Ponseti method.

**Methods:**

We performed a systematic review of comparative studies in which the cast change interval was varied. Scientific databases were searched for relevant publications, screened for eligibility and assessed for a risk of bias. A 'best evidence' synthesis tool was used to synthesize the results of the included studies and draw conclusions from relevant clinical outcomes.

**Results:**

Nine papers matched the inclusion criteria, which provided data of 587 subjects who had a total of 870 clubfeet. There is strong evidence for a positive relation between cast change interval and treatment duration. However, there is no evidence for any relation between the cast change interval and the required number of casts, tenotomy rate, required surgery or failure rate.

**Conclusions:**

Accelerated versions are as effective and safe as the traditional Ponseti method. However, more research is needed to assess the long-term results and to identify an optimal cast change interval.

## Introduction

Clubfeet are commonly treated with serial casting, according to the Ponseti method [1]. In this method, the clubfoot is manipulated and fixated in a corrected position using a plaster cast. This cast is typically changed every week. Occasionally, the cast is changed more frequently in an attempt to minimize the treatment duration (e.g. [2]). In low-income countries this can be especially important if practitioners are scarce and patients live far away from the hospital.

The original papers on the Ponseti method state that a new cast should be applied at four to seven [1] or five to seven-day intervals [3]. However, the time period is not motivated here nor have we found solid motivation elsewhere in literature. The reason for having a weekly treatment interval seems to be largely pragmatic. Clustering patients on one fixed day of the week is convenient for hospital planning and makes it easier to organize the necessary care around a newborn. Additionally, accommodating all clubfoot patients on a single day gives parents the opportunity to share their concerns with other parents [4]. Shortening the interval time could be advantageous for several reasons. Parents who do not have ready access to treatment facilities may have to leave home for the treatment duration. For those who can make outpatient visits to a clinic, shortening the treatment time will reduce the time during which their family life is interrupted. However, shortening the treatment time can only be done if a shorter interval is not detrimental to treatment outcome, if the number of hospital visits does not increase, and if no additional discomfort or pain is caused to the children by increasing the rate of correction. With this review we want to assess the influence of a shorter cast change interval.

When looking at the application of serial casting to stretch soft tissue in other disorders than clubfoot, we have found inconclusive or even contradictory evidence for the optimal cast change interval. In hand therapy, this is suggested to be two days [5]. However, removing the cast of a contracted finger after three days resulted in an increase in range of motion of 3.0° whereas removing the cast after six days resulted in an increase of 5.2° [6]. When the cast change interval was changed in the treatment of elbow, knee, wrist and ankle contractures, it was found that an interval of 1–3 days resulted in a shorter treatment with fewer complications when compared to an interval of 5–7 days [7]. As the underlying mechanisms causing these contractures might differ from the more complex clubfoot, the question remains how these findings on cast change intervals apply to the treatment of clubfoot.

The current systematic review compares the results from applying accelerated versions of the Ponseti method to the results of weekly cast changes to investigate the influence of the cast change interval on treatment outcomes.

## Materials and methods

A systematic review was performed on the existing literature dealing with the Ponseti method regarding the influence of cast change interval on treatment outcomes.

### Search protocol

A PRISMA-driven [8] systematic search of the PubMed, COCHRANE, WebOfKnowledge, Scopus, PeDRO, CINAHL and Google Scholar databases was conducted in October 2017 to identify relevant papers published between January 2005 and October 2017. The used search string was “Ponseti AND clubfoot AND duration AND cast* AND (Pirani OR Dimeglio)”. Reference lists of the full-texts retrieved for eligibility were screened to identify further relevant studies.

### Eligibility criteria and study selection

Comparative studies in which the cast change interval was varied were included. Full-text papers needed to be available written in English, German, French or Dutch. Exclusion criteria were conference abstracts, meta-analyses or review papers. Additional exclusion criteria were studies on non-idiopathic clubfoot, modifications to the original Ponseti method other than to the cast change interval and studies without a control group. Two reviewers (RBG, MCvdS) independently assessed the relevance of the identified papers based on the title and abstract. In a second stage, full-text papers were checked against inclusion and exclusion criteria (by RBG and MCvdS). Any doubts about eligibility were resolved by discussions between the two reviewers.

### Data extraction

Each selected paper was reviewed (by RBG) to extract relevant patient data, cast change interval, number of casts, treatment duration, required surgery, relapse rate and failure rate. Attempts were made to contact the authors of each selected paper for clarification and to access the raw data for a deeper analysis of the presented results. For each paper, this data was extracted for both the normal group (weekly cast changes) and the accelerated group (shortened interval). Complications were extracted as defined by the selected paper. Treatment duration was defined as the time from the application of the first cast until the removal of the final cast prior to the Achilles tenotomy. Required surgery was defined as any form of surgery after Ponseti treatment, including re-tenotomy. As defined by the Iowa Group, relapse was considered to be the re-appearance of any of the components of the deformity, including cavus, adductus, varus, and equinus [9]. Failure was defined as a post-cast-treatment Pirani score higher than 1.0, as defined by others [2, 10, 11].

### Quality assessment

The selected papers were independently assessed by the two reviewers (RBG, MCvdS) using Cochrane Collaboration’s tools for assessing risk of bias [12], scoring the papers with a ‘+’, ‘?’ or ‘-’. Criteria used were selection bias (1—randomization of groups, 2—comparability of both groups), attrition bias (3—sufficiency of follow-up, 4—definition of treatment outcomes), reporting bias (5—documentation of treatment outcomes), detection bias (6—blinded measurement of treatment outcomes) and performance bias (7 –blinded participants and personnel). Disagreements were solved during a consensus meeting. To be classified as low risk of bias, items 1, 2, 4 and 5 of the quality assessment needed to be scored as positive.

### Qualitative synthesis

Pooling of the data was considered impossible due to the clinical differences of the included studies and differences in reporting methods. Therefore, a ‘best evidence’ synthesis was performed as qualitative synthesis of the results. Based on the system used by [13], the ranking of levels of evidence was used from the method formulated by [14] (Table 1).

**Table 1:** Levels of evidence.

Level	Description
Strong evidence	Two or more studies with low risk of bias and by generally consistent findings in all studies (≥75% of the studies reported consistent findings)
Moderate evidence	One low risk of bias study and two or more high risk of bias studies and by generally consistent findings in all studies (≥75%)
Limited evidence	One or more high risk of bias studies or one low risk of bias study and by generally consistent findings (≥75%)
Conflicting evidence	Conflicting findings (<75% of the studies reported consistent findings)
No evidence	No studies could be found

### Statistical analysis

If statistical testing had not been performed in the original paper but the available extracted data allowed for it, differences in surgery, relapse and failure rate were tested for statistical significance using the two-tailed χ^2^ test or Fisher’s exact test. Statistical significance was defined as p < 0.05.

## Results

**Fig 1:**
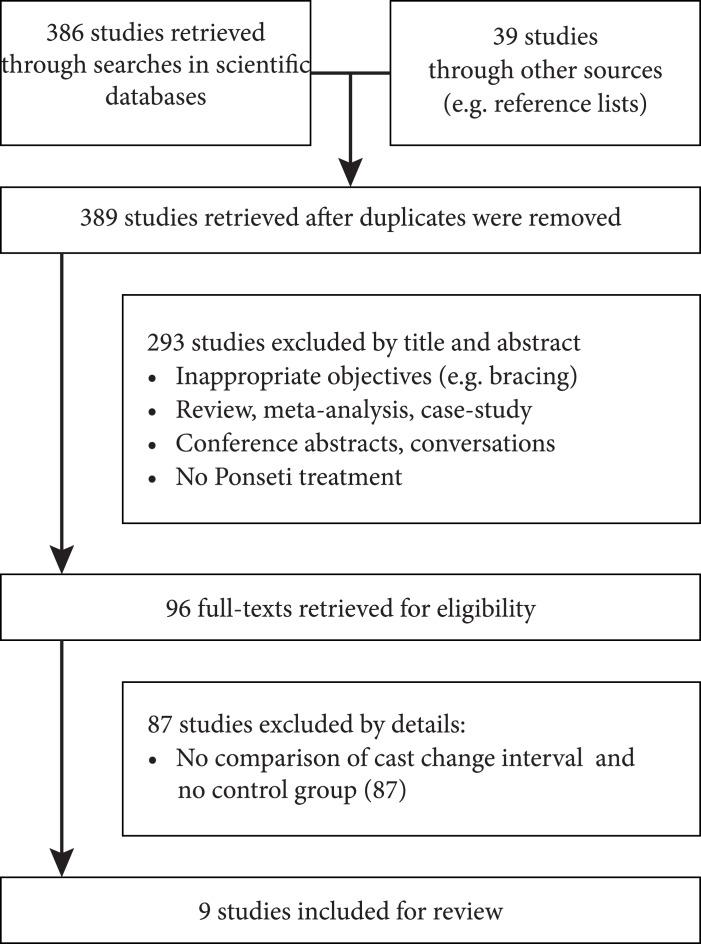
PRISMA flow diagram. Process of study identification and selection for outcome analysis [8].

The abstracts of 389 papers were screened for relevance. A total of 96 papers were found to contain relevant information, nine of which violated none of the exclusion criteria [2, 9–11, 15–19]. Fig 1 shows the flow diagram of the papers.

### Study characteristics

Characteristics of the selected papers are presented in Table 2, and these papers cover the treatment of a total of 587 subjects with a total of 870 clubfeet.

**Table 2:** Study characteristics.

Study	Study design	Group	Nr of Subjects	Nr of Clubfeet	Age at onset (months)	Prior treatment (%)	Pre-treatment Pirani score	Follow-up (months)
**Elgohary** (2015)	Prospective randomized	N	20	34	2.5	11.8	5.17	25.25
		A	21	32	2.7	15.6	5.13	23.38
**Gilani** (2014)	Prospective randomized	N	40	61	5.09	0 ^b^	4.12	-
		A	40	62	4.57		4.35	
**Harnett** (2011)	Prospective randomized	N	21	32	1.0	-	5.0 ^a^	8.0
		A	19	29	0.7		5.5 ^a^	8.5
**Ibraheem** (2017)	Prospective randomized	N	14	23	1.1	0 ^b^	5	-
		A	14	22	1.9		4.8	-
**Mageshwaran** (2016)	Prospective randomized	N	20	26	0.93	-	4.97	3 or 6
		A	20	25	0.92		5.025	3 or 6
**Morcuende** (2005)	Retrospective non-randomized	N	111	162	5	72 ^c^	-	-
		A	108	157	3			
**Sahu** (2015)	Prospective randomized	N	27	40	1.3	0 ^b^	5.03	11 ^c^^,^^e^
		A	26	40	1.21		5.3	
**Sharma** (2016)	Prospective randomized	N	20	26	0.75	0 ^b^	5.32	7.7
		A	20	27	0.77		5.21	8.2
**Xu** (2011)	*Unknown*, non-randomized	N	20	32	2.10	-	4.0 ^d^	48 ^c^
		A	26	40	3.09		4.1 ^d^	

N = Normal group, A = Accelerated group. Unless indicated otherwise, data is presented as it is in the selected paper, as means. Not reported data is indicated with ‘-’.

^a^ presented as median

^b^ patients with any form of prior treatment were excluded

^c^ for both groups combined

^d^ calculated over presented data

^e^ personal communication

#### Quality assessment

The results of the risk of bias analysis are presented in Table 3. Three studies were classified as having a low risk of bias [2, 11, 15]. The other six were classified as having a high risk of bias [9, 10, 16–19].

**Table 3:** Risk of bias analysis.

Study	1—Randomization of groups	2—Comparability of groups	3—Follow-up sufficiency	4—Definition of outcomes	5—Documentation of outcomes	6—Blinded assessment of outcomes	7—Blinded participants and personnel	Risk of bias
**Elgohary** (2015)	+	+	?	+	+	?	-	Low
**Gilani** (2014)	+	?	-	-	-	?	-	High
**Harnett** (2011)	+	+	?	+	+	?	-	Low
**Ibraheem** (2017)	+	+	-	+	?	-	-	High
**Mageshwaran** (2005)	+	+	-	?	+	?	-	High
**Morcuende** (2005)	-	-	?	+	-	?	-	High
**Sahu** (2015)	+	+	-	+	?	?	-	High
**Sharma** (2016)	+	+	-	+	+	?	-	Low
**Xu** (2011)	-	?	+	+	+	?	-	High

‘+’ was given if the used methodology was clear and adequate, and all required data was present

‘?’ was given if the used methodology was unclear or statistical information was missing

‘-’ was given if the used methodology was faulty or data was missing or not presented per subgroup

Note that in order to be classified as low risk of bias, items 1, 2, 4 and 5 needed to be positively scored

### Qualitative synthesis

The extracted data from the selected papers is presented in Table 4. The only surgery, relapse or failure rate data that scored positively when tested for a statistically significant difference was the number of relapses in Morcuende, Abbasi [9], as had already been reported in the original paper. The results of the statistical tests are presented in S1 File.

**Table 4:** Extracted data from the selected papers.

Study	Group	Interval (days)	Average nr of casts	Duration (days)	Complications	Tenotomy rate (%)	Failure rate (Pirani > 1.0) (n(%))	Surgery (n(%))	Relapse (n(%)
**Elgohary**	N	7	4.88	**33.36**	0 ^b^	91.2	0	3 (9%)	5 (15%)
(2015)	A	3.5	5.16	**18.13**		93.8	0	3(9%)	5 (16%)
**Gilani**	N	7	5.2	**36.4** ^d^	-	71.2 ^b^	2 (5%)	2 (5%)	-
(2014)	A	3.5	5.12	**17.92** ^d^			4 (10%)	4 (10%)	
**Harnett**	N	7	5 ^a^	**42**	0 ^b^	52	2 (10%)	2 (10%)	0 ^b^
(2011)	A	2.3	5 ^a^	**16**		79	3 (16%) ^e^	1 (5%)	
**Ibraheem**	N	7	5.26	52	-	96	0	-	-
(2017)	A	3.5	6.23	39		100	0		
**Mageshwaran**	N	7	5.55	52.8	0 ^b^	11.5	-	0	3 (15%)
(2016)	A	3.5	5.95	39.65		24		1 (5%)	4 (20%)
**Morcuende**	N	7	4 ^b^	**24**	-	81	-	21 (10%) ^b^	**25 (23%)**
(2005)	A	5		**16**		85			**11 (10%)**
**Sahu**	N	7	6.2 ^c^	**57.4** ^c^	-	78	-	0	9 (23%)
(2015)	A	3	7.4 ^c^	**23.8** ^c^		83		1 (4%)	13 (33%)
**Sharma**	N	7	5.08	**35.24**	0 ^b^	77	1 (5%)	1 (5%)	0 ^b^
(2016)	A	3.5	4.15	**14.19**		74	1 (5%)	1 (5%)	
**Xu**	N	7	5.25	**35.35**	0 ^b^	87.5	5 (16%)	4 (13%)	-
(2011)	A	3.5	5.04	**20.61**		87.5	6 (15%)	6 (15%)	

N = Normal group, A = Accelerated group. Unless indicated otherwise, data is presented as it is in the selected paper. Not reported data is indicated with ‘-’. Numbers in **bold** represent a statistical significant difference (p < 0.05).

^a^ presented as median

^b^ for both groups combined

^c^ including tenotomy cast

^d^ calculated from casts × interval, not included in the best evidence synthesis

^e^ three subjects crossed-over to the control group because they still had Pirani > 1.0 after 21 days

Only negligible differences—if any—in terms of required number of casts, tenotomy rate, required surgery or failure rate were reported between the groups in the selected articles (Table 4). The best evidence synthesis revealed a strong evidence for the absence of a relation between cast change interval and these clinical outcomes (Table 5). Five of the selected studies report that no (short-term) complications were observed in either group [2, 11, 15, 17, 19], and the remaining studies did not report on complications.

**Table 5:** Best evidence synthesis of outcome measures.

	Nr of studies	Statistically significant difference	No statistically significant difference	Best evidence synthesis	Comments
**Average nr of casts**	7	LR: 0 HR: 0	LR: 3 HR: 4	Strong evidence no relation	Statistical significance not reported in Sahu, Rajavelu (18). Mean 6.2 (range 4–10) vs 7.4 (5–10) casts: inconclusive No statistical test in Gilani, Ahmed (10). Mean±SD 5.2±1.62 vs 5.12±1.53 casts: assumed as no statistical significant difference.
**Tenotomy rate**	8	LR: 0 HR: 0	LR: 3 HR: 5	Strong evidence no relation	
**Duration**	6	LR: 3 HR: 3	LR: 0 HR: 0	Strong evidence positive relation	Statistical significance not reported in Sahu, Rajavelu (18). Mean 57.4 vs 23.8 days: inconclusive
**Surgery**	7	LR: 0 HR: 0	LR: 3 HR: 4	Strong evidence no relation	Morcuende, Abbasi (9) surgery data for both groups combined: inconclusive
**Relapse**	4	LR: 0 HR: 1	LR: 1 HR: 2	Moderate evidence no relation	
**Failure** **(Pirani > 1.0)**	5	LR: 0 HR: 0	LR: 3 HR: 2	Strong evidence no relation	No statistical test in Elgohary and Abulsaad (15). No difference in post-treatment Pirani score (reported p = 0.89), all scores were ≤ 1.0: assumed as no statistical significant difference.

LR = Low Risk of bias, HR = High Risk of bias

“Significant difference” indicates the number of selected studies in which a statistically significant difference was found.

#### Relapse

The best evidence synthesis gave moderate evidence for the absence of a relation between cast change interval and relapse (Table 5). Morcuende, Abbasi [9] report a significant difference in number of relapses (11/108 in the accelerated group vs 25/111 in the traditional group, *p* = 0.01) while others did not report such an observation. The division of groups in this study was not random but based on geographical location. The only study [19] with sufficiently long follow-up did not report on long-term results such as relapse. Those that did report on relapse rate had either a short [2, 11, 15, 17, 18] and/or poorly-defined [9, 18] follow-up.

#### Treatment duration

There is strong evidence for a relation between cast change interval and treatment duration, but not for the number of casts required to correct clubfoot. Since a shorter interval decreases the time per cast, but the total number of casts does not change, the treatment duration is significantly shorter in the accelerated groups of all studies.

## Discussion

An increasing number of studies continues to be published in which the Ponseti method’s interval is shorter than the traditional week. Only few studies use a control group to investigate the influence of the cast change interval on treatment outcomes. Individually, the selected nine studies were able to show the feasibility of an accelerated Ponseti method, but could not identify clear differences in clinical treatment outcomes. The current systematic review attempts to present additional conclusive evidence based on a best evidence synthesis, and to identify missing information.

Overall, this review suggests that the accelerated versions of the Ponseti method are in fact as effective as the traditional method in the initial correction of idiopathic clubfoot. The shorter cast change intervals cause a decreased treatment duration without deteriorating clinical outcome.

None of the other selected studies can confirm the conclusion of Morcuende, Abbasi [9] who, based on anecdotal observations only, suggest that a 5-day interval is the shortest safe interval. In line with this, cohort studies involving weekly cast-changes report approximately 20% of patients experiencing short-term complications such as blisters and skin problems, compared to 16% of those undergoing a cast-change twice a week [20–22]. However, insufficient follow-up was available in the selected papers. As such, this review could not address any possible problems that might arise during the bracing period, for example brace compliance. Furthermore, long term results of the accelerated Ponseti method including proper relapse rate and functional outcome remain unknown.

The advantage of a shorter treatment duration is for the caregivers of the patients. Especially caregivers in low-income countries with limited access to healthcare centers are likely to benefit from a shortened casting period [2, 11, 16, 23]. Other authors suggest that benefits of a shorter treatment are the reduced risk of skin problems, cast slipping and osteopenia [2, 11].

### Limitations

Since the outcome of the Ponseti method is affected by factors such as experience [24] or the strictness of the adherence to the Ponseti method [25], treatment outcomes such as the required number of casts vary among health care institutions. A form of bias would be introduced if results from different institutions would be compared. We have therefore deliberately chosen to only include controlled trials. Inevitably, this choice has limited the selected number of studies which—in combination with the heterogeneity in research methodology and data reporting—did not allow for any quantitative meta-analysis within this systematic review and therefore we performed a qualitative best-synthesis analysis.

Part of the best-synthesis analysis method is to determine when a study is classified as low risk. Since, in our opinion, it is practically impossible to blind participants and personnel involved from the cast change interval used during the treatment, and the outcome measures were mostly objective (e.g. whether or not tenotomy or surgery was performed), we excluded these items from the classification of low/high risk of bias. Non-blinding during the assessment of treatment outcomes in follow-up might have led to performance and detection bias but we assumed this to be minimal. Insufficient or poorly defined follow-up increases the risk of attrition bias, but this domain was excluded from the judgement of risk since mostly short-term outcome measures were extracted (e.g. treatment duration, casts, tenotomy). The long-term effects of shorter cast change intervals remain poorly documented. Would we have weighted all items of bias similarly, this would have lowered the level of evidence to limited for all outcome measures as no study would be classified as low risk of bias. Future studies on cast change intervals should aim to reduce possible risks of bias to a minimum and clearly state how these risks were minimized.

As is a problem in many fields of research, researchers who achieved poor results might have refrained from publishing them. Therefore, this risk of publication bias might have caused the best evidence synthesis in this systematic review to be too optimistic.

For practical reasons, we have limited our search to the languages English, German, French and Dutch, and to the translated abstracts of leading journals in other languages which we found during our search.

### Optimal cast change interval

According to the best evidence synthesis, no evidence exists to support the use of a cast change interval of one week. The combined results of the best evidence synthesis in this report can be used to suggest some fundamental principles for the correction of a clubfoot. One scholar mentioned that *“the tissues might need some time in the corrected position in the cast to be able to adapt through this growth and change”* [26]. If the adaptation time were to be longer than the used cast change interval (e.g. 5 days versus half a week), then the accelerated method would only achieve partial adaptation. Such incomplete adaptation would have resulted in adverse treatment outcomes (for example, more casts, more complications and more surgery). However, none of the studies made such observations, which implies that the tissues might need less than a third of a week to adapt, as shown by Harnett, Freeman [2]. As long as treatment outcomes do not worsen as a result of increasingly shorter cast change intervals, the interval apparently still exceeds the required adaptation time, and there is still margin for further acceleration of the treatment process.

Elgohary and Abulsaad [15] asked the question *“what is the least time interval between castings to be applied safely*?*”*. From the numbers available, we are unable to provide a conclusive answer to that question, but it does seem that the limit has not yet been reached. To the best of our knowledge, the shortest reported interval can be found in a case study by Sutcliffe, Vaea [27]. After four casts in one week, the Pirani scores of two young patients were 1.5 and 3.5. In this case study the correction was still insufficient and more casts might have been required to achieve optimum correction. Anecdotal information exists for even shorter intervals with even daily cast changes. Cummings, Davidson [28] mention that *“More rapid correction has been achieved with more frequent (daily) cast changes and manipulation”*, but without going into detail. A large randomized controlled trial with multiple cast change intervals (weekly, twice per week, daily, etc.) with adequate follow up is necessary to determine the optimal cast change interval.

### Mechanobiology

What the shortest acceptable time interval is might be ascertained by considering theories from the discipline of mechanobiology. Much of the knowledge about the viscoelastic behavior of biological materials is based on experiments using cadaver material, such as rat tail tendons [29]. When subjected to a constant strain, the resulting stress reduces over time, known as stress-relaxation. The stresses in dead collagen fibers obtained from rat tail tendons reached equilibrium within several minutes [29, 30].

However, little is yet known about the combined behavior of stress-relaxation, tissue remodeling and growth of living biological tissue. During research into limb lengthening, it was observed that most of the relaxation occurs within the first two hours after distraction [31]. In serial casting of contracted elbows, knees and ankles, it was observed that the tension within the cast dropped by 80% within the first 24 hours [32]. A high initial pressure was measured underneath the Ponseti cast although pressure sores are rarely observed, which suggests a rapid decrease of this pressure resulting from a short adaptation time [33]. Preliminary cast / clubfoot interface pressure measurements by one of the authors suggest that the tissues may have reached full adaptation just several hours after casting.

## Conclusions

There is strong evidence that accelerated versions of the Ponseti method can safely be used in the treatment of clubfoot without risking any increase in the required number of casts, the failure rate or the surgery rate. Shorter intervals significantly decrease treatment duration, which means that for each patient the most convenient duration may be selected. More research is needed to determine any existing optimal cast change interval and to investigate the long-term effects of shorter cast change intervals.

## Supporting information

S1 File Supplementary data file. The excel file contains all steps of the search and analysis presented in this manuscript and more.
(XLSX)

S2 File PRISMA 2009 flow diagram. The word file contains the PRISMA flow diagram.
(DOC)

S3 File PRISMA 2009 checklist. The word file contains the PRISMA 2009 checklist.
(DOC)
