# Supplementary material for: Influence of cast change interval in the Ponseti method: A systematic review
Source: PLoS One. 2018 Jun 22;13(6):e0199540. doi: 10.1371/journal.pone.0199540 (PMC6014642; doi:10.1371/journal.pone.0199540)
Supplement: S2 File — The word file contains the PRISMA flow diagram. (DOC) [file pone.0199540.s002.doc]

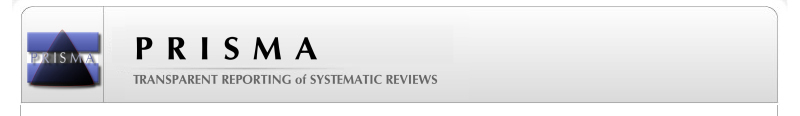
**PRISMA 2009 Flow Diagram**

**Screening**

**Included**

**Eligibility**

**Identification**

Records identified through database searching
(n = 386)

Additional records identified through other sources
(n = 39)

Records after duplicates removed
(n = 389)

Records screened
(n = 389)

Records excluded
(n = 293)

Full-text articles assessed for eligibility
(n = 96)

Full-text articles excluded, with reasons
(n = 87)

Studies included in qualitative synthesis
(n = 9)

Studies included in quantitative synthesis (meta-analysis)
(n = 9)
